# Diagnostic and Prognostic Value of External Anal Sphincter EMG Patterns in Multiple System Atrophy

**DOI:** 10.1002/mds.28938

**Published:** 2022-02-04

**Authors:** Massimiliano Todisco, Giuseppe Cosentino, Serena Scardina, Mauro Fresia, Paolo Prunetti, Antonio Pisani, Enrico Alfonsi

**Affiliations:** ^1^ Translational Neurophysiology Research Unit IRCCS Mondino Foundation Pavia Italy; ^2^ Movement Disorders Research Center IRCCS Mondino Foundation Pavia Italy; ^3^ Department of Brain and Behavioral Sciences University of Pavia Pavia Italy; ^4^ Department of Biomedicine, Neuroscience and advanced Diagnostics (BIND) University of Palermo Palermo Italy

**Keywords:** diagnosis, EMG, multiple system atrophy, Parkinson's disease, prognosis

## Abstract

**Background:**

It is debated whether external anal sphincter (EAS) electromyography can distinguish between multiple system atrophy (MSA) and Parkinson's disease (PD), whereas its usefulness for MSA prognosis is unknown.

**Objectives:**

We explored the diagnostic and prognostic value and clinical correlations of EAS electromyography patterns in MSA.

**Methods:**

We collected clinical data and EAS electromyography findings in 72 patients with MSA and 21 with PD.

**Results:**

We identified four EAS patterns. The normal pattern was frequently observed in PD and associated with prolonged survival when identified in MSA. Abnormal patterns were predominant in MSA. The most severe pattern was associated with the highest likelihood of MSA diagnosis and with the worst prognosis in the MSA cohort. MSA patients with EAS abnormalities often showed urogenital symptoms and fecal incontinence.

**Conclusions:**

The increasing severity of EAS electromyography patterns paralleled diagnostic accuracy and survival in MSA, and correlated with prevalence of bladder and bowel symptoms. © 2022 The Authors. *Movement Disorders* published by Wiley Periodicals LLC on behalf of International Parkinson and Movement Disorder Society

Multiple system atrophy (MSA) is a sporadic neurodegenerative disorder characterized by autonomic failure and parkinsonian or cerebellar syndrome.^1^ Early‐onset severe autonomic symptoms diminish the quality of life and are associated with poor prognosis in MSA.^2^, ^3^, ^4^, ^5^, ^6^, ^7^, ^8^ A prompt diagnosis is therefore crucial to prevent potentially life‐threatening complications. However, the clinical presentation of MSA sometimes overlaps with the phenomenology of Parkinson's disease (PD), especially in MSA patients showing levodopa‐responsive parkinsonism without overt cerebellar involvement in the early stages.^9^ Evidence that PD patients can also show autonomic symptoms further complicates the diagnostic picture.^10^, ^11^, ^12^

Several authors suggested that electromyography (EMG) of the external anal sphincter (EAS) may help in the differential diagnosis, particularly within the first 5 years of symptom onset.^13^, ^14^, ^15^, ^16^, ^17^, ^18^, ^19^, ^20^, ^21^, ^22^, ^23^ Indeed, in contrast with normal findings in PD patients, most subjects with MSA show EAS neurogenic abnormalities that are considered an electrophysiological correlate of Onuf's nucleus degeneration, a pathological hallmark of MSA.^24^ Despite the suggestion to include EAS EMG in the diagnostic workup of MSA,^25^ some authors questioned its diagnostic value,^26^, ^27^, ^28^, ^29^ and current diagnostic criteria do not acknowledge this investigation as part of the instrumental toolbox.^1^

Here, we identified four EAS EMG patterns, aiming to explore their usefulness in the differential diagnosis between MSA and PD, their association with clinical features, and their role as prognostic predictors in MSA.

## Patients and Methods

### Study Design

In this retrospective study, we consecutively enrolled 72 patients with probable MSA and 21 with PD between 2003 and 2019. The local ethics committee approved the study, and participants gave written informed consent. MSA was diagnosed according to current international criteria,^1^ and PD in accordance with the Movement Disorder Society criteria.^30^ Diagnosis was ascertained at last available follow‐up. We applied the following exclusion criteria: history of lumbosacral radiculopathy, lumbar spinal stenosis, pelvic irradiation, lumbar spine or pelvic surgery; diagnosis of diabetes mellitus, polyneuropathy, pudendal nerve injuries, cauda equine or conus medullaris syndrome; presence of severe hemorrhoids or previous hemorrhoidectomy. Diagnosis was unclear at hospital admission, during which patients underwent clinical and instrumental evaluations, including brain magnetic resonance imaging and EAS EMG, routinely performed at our institute to assist the diagnostic process. MSA patients or their caregivers were then contacted by phone, allowing us to ascertain the survival times of 49 MSA patients who died by 2021.

### Clinical Assessment

Motor impairment in MSA patients was assessed by means of the Unified Multiple System Atrophy Rating Scale, motor section (UMSARS II). Levodopa equivalent daily dose (LEDD) was calculated. The parkinsonian (MSA‐P) and cerebellar (MSA‐C) variants of MSA were identified based on the predominant motor phenotype. We also established the symptom type at disease onset and the presence of urogenital symptoms or fecal incontinence at hospital admission. Urogenital symptoms included storage disturbances (ie, urinary urgency or incontinence), voiding disorders (ie, incomplete bladder emptying or urinary retention), and erectile dysfunction in males. Orthostatic symptoms meant disturbances deriving from orthostatic hypotension, such as dizziness or blurred vision only on standing. Motor disturbances referred to symptoms due to parkinsonian or cerebellar syndrome.

### EMG Investigation

Detailed EMG description is reported in Appendix S1. Based on presence and severity of the underlying neurogenic damage, we identified four EAS EMG patterns (Fig. S1):

- no pathological spontaneous activity, normal duration and recruitment of motor unit action potentials (MUAPs) (normal findings);
- neurogenic MUAPs, normal recruitment of MUAPs, with/without pathological spontaneous activity (mild neurogenic damage);
- neurogenic MUAPs, reduced recruitment of MUAPs, with/without pathological spontaneous activity (moderate neurogenic damage);
- absent recruitment of MUAPs, with/without pathological spontaneous activity (severe neurogenic damage).

### Statistical Analysis

Patients' groups were compared using the χ^2^ test, *t* test, or Wilcoxon rank‐sum test. Differences based on EAS EMG patterns in the MSA cohort were explored by means of the χ^2^ test or Kruskal‐Wallis test. Bonferroni correction for multiple comparisons was applied. Nominal logistic regression analyses were performed to test whether EAS EMG patterns were diagnostic predictors. Receiver operating characteristic curves and odds ratio (OR) values were then calculated. Survival was defined as time from symptom onset to death. Differences in survival based on EAS EMG patterns were explored by means of the Kruskal‐Wallis test and post‐hoc Dunn's test. Survival analysis was carried out using Kaplan–Meier curves and log‐rank test to compare EAS EMG patterns. Statistical significance was set at *P* < 0.05. JMP Pro 14.0 software was used for statistical analyses.

## Results

**FIG 1:**
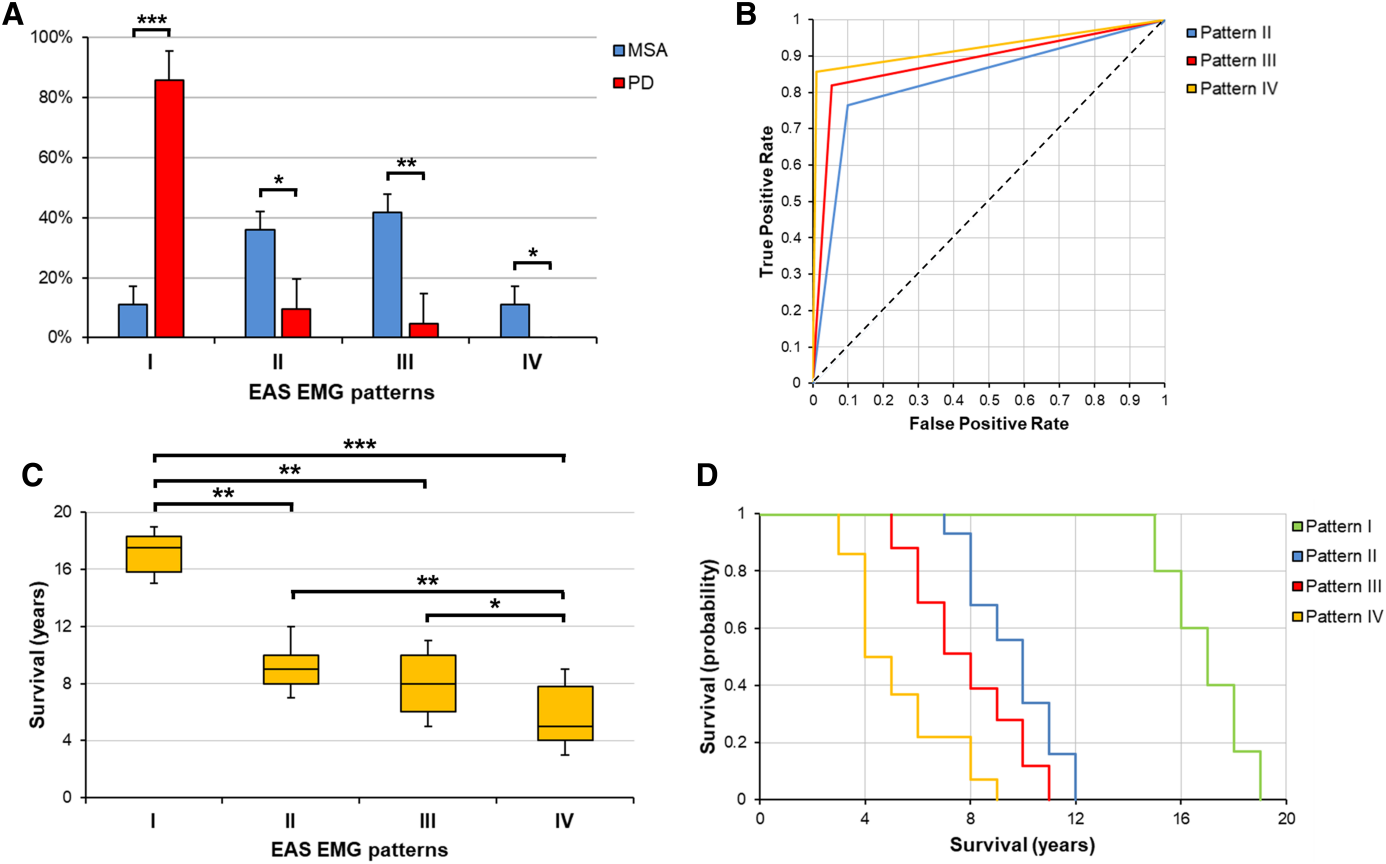
**(A)** Frequency distribution of EAS EMG patterns in MSA and PD patients. Vertical error bars represent standard errors. Horizontal bars indicate significant differences detected with the χ^2^ test (*P < 0.05, **P < 0.01, ***P < 0.001). **(B)** Receiver operating characteristic curves of EAS EMG patterns for differential diagnosis between MSA and PD. MSA diagnosis was used as a positive level for logistic regression analyses. Pattern II showed an area under the curve (AUC) of 0.83, sensitivity of 76.5%, specificity of 90.0%, positive predictive value of 92.9%, and negative predictive value of 69.2%. Pattern III showed an AUC of 0.88, sensitivity of 82.0%, specificity of 94.7%, positive predictive value of 96.8%, and negative predictive value of 74.5%. Pattern IV showed an AUC of 0.96, sensitivity of 85.6%, specificity of 99.0%, positive predictive value of 99.5%, and negative predictive value of 89.7%. **(C, D)** Survival differences by EAS EMG patterns in MSA patients. **(C)** Horizontal bars indicate significant differences detected with the post‐hoc Dunn's test after the Kruskal‐Wallis test (*P < 0.05, **P < 0.01, ***P < 0.001). **(D)** Kaplan–Meier curves for each EAS EMG pattern. EAS, external anal sphincter. [Color figure can be viewed at wileyonlinelibrary.com]

MSA and PD patients did not differ regarding gender, age at symptom onset, age and disease duration at EMG, or LEDD (Table S1). The distribution of EAS EMG patterns was found to differ between the two cohorts (*P* < 0.001): pattern I was more frequent in subjects with PD as compared with MSA patients (85.7% vs. 11.1%, respectively, *P* < 0.001), whereas each of the abnormal patterns was more frequent in the MSA group (pattern II: 36.1% in MSA vs. 9.5% in PD, *P* = 0.011; pattern III: 41.7% in MSA vs. 4.8% in PD, *P* = 0.002; pattern IV: 11.1% in MSA vs. no PD patient, *P* = 0.028) (Fig. 1A). Differences persisted after excluding MSA‐C patients.

**FIG 2:**
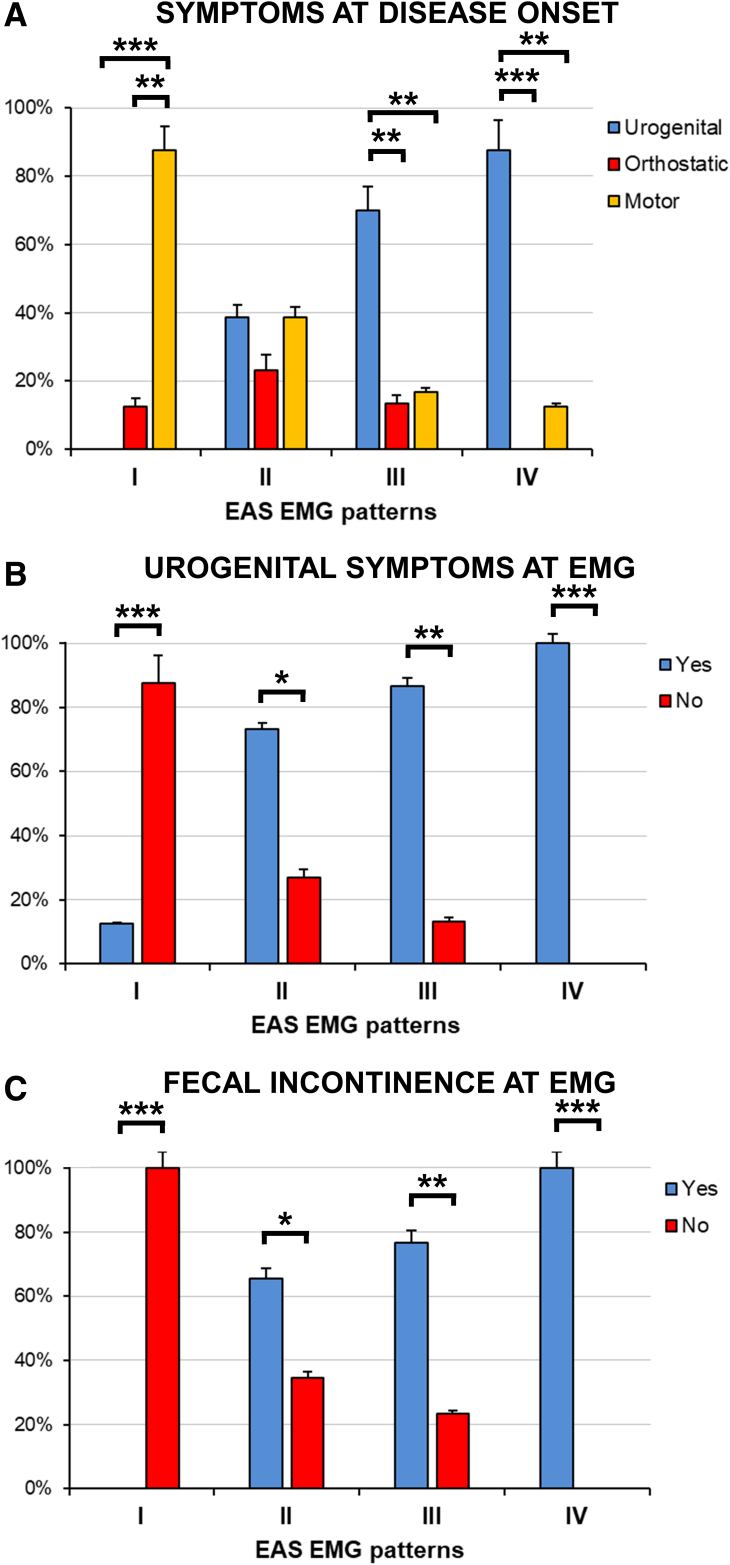
Frequency distribution of symptom types at disease onset **(A)** and at hospital admission **(B, C)** based on EAS EMG patterns in MSA patients. Vertical error bars represent standard errors. Horizontal bars indicate significant within‐pattern differences detected with the χ^2^ test (*P < 0.05, **P < 0.01, ***P < 0.001), whereas between‐pattern comparisons are reported in Appendix S2. **(A)** The predominance of urogenital symptoms at disease onset in subjects with patterns III or IV, versus the group with pattern I, was significant. **(B, C)** Urogenital symptoms and fecal incontinence at hospital admission were more frequent in patients with patterns II, III, or IV with respect to subjects with pattern I, and in patients with pattern IV as compared with subjects with pattern II. EAS, external anal sphincter. [Color figure can be viewed at wileyonlinelibrary.com]

The presence of an abnormal pattern correlated with MSA diagnosis (*R* ^*2*^ = 0.43, *P* < 0.001), with an area under the curve of 0.87, sensitivity of 88.9%, specificity of 85.7%, positive predictive value of 95.5%, negative predictive value of 69.2%, and OR of 48.0 (95% confidence interval [CI]: 11.5–199.8). The likelihood of MSA diagnosis paralleled the severity of EAS EMG impairment (Fig. 1B). Pattern II was a diagnostic predictor of MSA (*R* ^*2*^ = 0.32, *P* < 0.001), with an OR of 29.3 (95% CI: 5.6–54.1). Pattern III correlated with MSA diagnosis (*R* ^*2*^ = 0.35, *P* < 0.001) and showed an OR of 67.5 (95% CI: 7.8–104.9). Pattern IV predicted MSA diagnosis (*R* ^*2*^ = 0.44, *P* < 0.001), with an OR of 103.7 (95% CI: 23.8–219.7). Demographic and clinical differences based on EAS EMG patterns in the MSA cohort are shown in Figure 2 and Appendix S2.

The following median survival times were recorded in MSA patients: 17.5 years (range: 15–19) in 6 patients with pattern I; 9 years (range: 7–12) in 16 subjects with pattern II; 8 years (range: 5–11) in 19 patients with pattern III; 5 years (range: 3–9) in eight subjects with pattern IV (Fig. 1C). As compared with the group with pattern I, survival was shorter in subjects with patterns II (*P* = 0.004), III (*P* = 0.002), or IV (*P* < 0.001). Patients with pattern IV also had worse prognosis than subjects with patterns II (*P* = 0.009) or III (*P* = 0.011). These findings were confirmed by Kaplan–Meier analyses, which showed poorer prognosis in patients with patterns II (vs. pattern I, *P* < 0.001), III (vs. pattern I, *P* < 0.001), or IV (vs. pattern I, *P* < 0.001; vs. pattern II, *P* = 0.001; vs. pattern III, *P* = 0.007) (Fig. 1D). Differences persisted after adjustment for disease duration at EMG.

## Discussion

We identified EAS EMG patterns of increasing severity, assessing the value of the resulting novel classification for differential diagnosis and prognostic stratification of MSA patients. EAS electrophysiological impairment was found to parallel diagnostic accuracy and survival: the more severe the EMG alterations, the greater the likelihood of MSA diagnosis, the worse the patient's prognosis. The pathophysiological relevance of EAS EMG patterns was corroborated by their association with symptom type at disease onset and with prevalence of bladder and bowel symptoms. MSA patients with EAS EMG abnormalities often showed fecal incontinence and urogenital symptoms, which were also frequently present at disease onset in subjects with impaired MUAP recruitment. Indeed, the disease process could begin in the sacral spinal cord before spreading to other regions responsible for motor impairment and cardiovascular autonomic dysfunction.^31^, ^32^ Conversely, MSA patients without EAS EMG alterations did not have fecal incontinence, rarely showed urogenital symptoms, and commonly presented with motor disturbances at disease onset.

Evaluation of MUAP recruitment allowed us to reveal a broader spectrum of EAS EMG abnormalities. Some authors subjectively graded MUAP interference pattern during maximal voluntary contraction, considering its reduction as an alternative expression of Onuf's nucleus degeneration in MSA.^19^, ^23^ Instead, in a small MSA cohort, Gilad and colleagues objectively assessed several recruitment parameters, such as the mean number of MUAPs per insertion site.^26^ Based on their observation of reduced recruitment pattern in the absence of other features of neurogenic damage, these authors hypothesized upper motor neuron degeneration rather than the loss of lower motor neurons of Onuf's nucleus in MSA patients. The reduced MUAP recruitment found in our study was invariably associated with increased MUAP duration, thus reflecting either a more severe Onuf's nucleus degeneration or a combination of lower motor neuron impairment and neuronal loss in supraspinal areas (eg, the pontine micturition and storage centers) that modulate Onuf's nucleus function.^33^, ^34^

We found high diagnostic accuracy of EAS EMG patterns in discriminating between MSA and PD, in keeping with studies analyzing single EMG parameters.^14^, ^18^, ^23^ The finding of lower sensitivity than specificity implies that, as seen in 11% of our cohort and in agreement with pathology evidence,^35^ some MSA patients do not show EAS EMG abnormalities, suggesting Onuf's nucleus preservation.

This study is the first to explore the prognostic value of EAS EMG alterations in MSA. Our results support previous clinical and urodynamic observations, since lower urinary tract symptoms and reduced detrusor contractility were shown to be among the strongest survival predictors in MSA.^5^, ^6^, ^7^, ^8^, ^36^ Neurogenic urinary dysfunction was linked to recurrent lower urinary tract infections, a primary cause of death in MSA.^37^ Moreover, urinary symptoms were associated with the loss of medullary serotonergic neurons, which contribute to micturition modulation and respiratory rhythmogenesis, increasing the risk of sudden death during sleep in MSA patients.^6^, ^38^, ^39^

Early severe Onuf's nucleus degeneration without significant progression over time could account for lack of association between EAS EMG patterns and disease duration in the MSA cohort, suggesting the usefulness of EAS EMG regardless of disease stage. However, the diagnostic and prognostic value of this investigation in the early stages should be thoroughly explored in future studies.

The retrospective design and possibility of misdiagnoses given the lack of neuropathological confirmation are limitations of this study. Furthermore, single‐MUAP analysis is time‐consuming and examiner‐dependent, but also the automated techniques require manual revision to ensure accurate calculation of MUAP duration.^17^, ^40^ In conclusion, the patterns described herein may be a valuable diagnostic and prognostic tool, and therefore EAS EMG should be recommended especially when the clinical picture is unclear. A normal EAS EMG pattern could identify a small MSA cohort characterized by less neurodegeneration and prolonged survival.

## Author Roles

1. Research project: A. Conception, B. Organization, C. Execution.
2. Statistical analysis: A. Design, B. Execution, C. Review and critique.
3. Manuscript preparation: A. Writing of the first draft, B. Review and critique.

M.T.: 1A, 1B, 1C, 2A, 2B, 3A

G.C.: 1C, 2C, 3B

S.S.: 1C, 2C, 3B

M.F.: 1C, 2C, 3B

P.P.: 1C, 2C, 3B

A.P.: 2C, 3B

E.A.: 1A, 1B, 1C, 2C, 3B

## Full financial disclosure for the previous 12 months

M.T. is part of Advisory Board for Zambon; he received honoraria from Zambon. Other authors declare no competing interests.

## Supporting information


**APPENDIX S1**. Supporting Information

**APPENDIX S2**. Supporting Information

**FIG. S1** Illustrative traces of EAS EMG patterns. In each panel, the upper traces show the presence or absence of pathological spontaneous activity, whereas the lower traces show motor unit action potential (MUAP) parameters (ie, duration and spatial recruitment). Pattern I: no spontaneous activity and normal MUAP parameters. Pattern II: spontaneous activity (fibrillation potentials and positive sharp waves), increased duration of MUAPs, and normal recruitment of MUAPs (three MUAPs per insertion site). Pattern III: spontaneous activity (fibrillation potentials and positive sharp waves), increased duration of MUAPs, and reduced recruitment of MUAPs (one MUAP per insertion site). Pattern IV: spontaneous activity (complex repetitive discharges) and absent recruitment of MUAPs. EAS, external anal sphincter.

**TABLE S1** Demographic and clinical features in MSA and PD patients

## Data Availability

Raw data used in this study are available in the Zenodo repository: https://doi.org/10.5281/zenodo.5775547
