# Supplementary material for: Diagnostic and Prognostic Value of External Anal Sphincter EMG Patterns in Multiple System Atrophy
Source: Mov Disord. 2022 Feb 4;37(5):1069–74. doi: 10.1002/mds.28938 (PMC9305564; doi:10.1002/mds.28938)
Supplement: Supplementary file 1 — APPENDIX S1. Supporting Information [file MDS-37-1069-s001.docx]

**EMG investigation**

EAS EMG in MSA and PD patients was performed by a neurologist with expertise in clinical neurophysiology, who was blinded to the diagnosis and other clinical findings. EMG was carried out using a Synergy SYN5-C EMG machine (Viasys Healthcare, Old Woking, Surrey, UK). Since bladder and rectal filling can influence EAS basal tonic activity,^1,2^ EMG was performed after bladder and bowel emptying. Patients were asked to lie on their left side, with their hips and knees flexed, while their right thigh was electrically grounded. A 28-gauge concentric needle electrode was inserted under audio guidance into the four quadrants of the EAS. For the superficial layer, the needle electrode was inserted, perpendicularly, 1 cm laterally to the anal orifice to a depth of 3–6 mm; and for the deep layer, it was inserted at the anal orifice at an angle of 30º to a depth of 15–25 mm.^1,3^ The following EMG parameters were evaluated: presence of pathological spontaneous activity (i.e., fibrillation potentials, positive sharp waves, or complex repetitive discharges); duration of MUAPs; and spatial recruitment of MUAPs. The bandpass filter was set at 3 Hz–10 kHz. With regard to the sensitivity settings, we used a gain of 50 μV/division and a sweep speed of 20 ms/division to assess spontaneous activity, and a gain of 100 μV/division and a sweep speed of 5 ms/division to analyze MUAPs. Given that pathological spontaneous activity can be difficult to distinguish from EAS basal tonic discharge,^4,5^ we evaluated it after asking patients to simulate defecation. Moreover, the characteristic shape and firing rate of pathological spontaneous activity can facilitate its detection, in the event of persistent activity of some MUAPs in patients who are unable to fully relax their EAS. We performed single-MUAP analysis by means of the ‘trigger and delay line’ technique, which makes it possible to identify MUAPs with the highest amplitude values.^6^ We analyzed MUAPs triggered during either basal tonic activity or a state of constant slight voluntary activation, in keeping with other authors.^1,3,7-9^ Despite the automatic positioning of cursors by the EMG software, manual revision was needed in order to ensure the correct placement of markers, delete duplicated MUAPs, and include satellite potentials (also known as late components of MUAPs) in the calculation of MUAP duration. This latter parameter has been shown to allow early and reliable discrimination of EAS neurogenic abnormalities in MSA.^4,5,10^ For each patient, the duration of 20 MUAPs was compared to reference values obtained from 40 age-matched healthy subjects (23 men; mean age ± SD: 61.3 ± 10.5 years; age range: 48–76 years). Normative values of mean duration ± SD were 6.8 ± 1.4 ms (range: 3.2–10.5). MUAPs were categorized as ‘neurogenic’ when their mean duration exceeded the 97.5^th^ percentile of normal range, corresponding to 10.2 ms, which is in line with the cut-off proposed by most previous studies.^4,5,10-16^ Spatial recruitment of MUAPs was measured as the mean number of MUAPs per insertion site during basal tonic activity and after voluntary activation. In agreement with other findings from multi-MUAP analysis,^14^ at least three MUAPs per insertion site could be detected in our group of healthy subjects; thus, recruitment was classified as ‘reduced’ when a mean number of less than three MUAPs per insertion site was identified. The entire EAS EMG investigation was repeated in a random sample of 20 patients by a second clinical neurophysiologist, again in a blinded fashion. In these subjects, the two examiners were in complete agreement with regard to the assignment of EAS EMG patterns.

**References**

1. Podnar S, Vodusek DB. Standardisation of anal sphincter EMG: high and low threshold motor units. Clin Neurophysiol 1999;110(8):1488–1491.
2. Podnar S, Mrkaić M, Vodusek DB. Standardization of anal sphincter electromyography: quantification of continuous activity during relaxation. Neurourol Urodyn 2002;21(6):540–545.
3. Podnar S, Rodi Z, Lukanovic A, Trsinar B, Vodusek DB. Standardization of anal sphincter EMG: technique of needle examination. Muscle Nerve 1999;22(3):400–403.
4. Palace J, Chandiramani VA, Fowler CJ. Value of sphincter electromyography in the diagnosis of multiple system atrophy. Muscle Nerve 1997;20(11):1396–1403.
5. Libelius R, Johansson F. Quantitative electromyography of the external anal sphincter in Parkinson's disease and multiple system atrophy. Muscle Nerve 2000;23(8):1250–1256.
6. Nandedkar SD, Barkhaus PE, Charles A. Multi-motor unit action potential analysis (MMA). Muscle Nerve 1995;18(10):1155–1166.
7. Podnar S, Vodusek DB, Stâlberg E. Standardization of anal sphincter electromyography: normative data. Clin Neurophysiol 2000;111(12):2200–2207.
8. Gilad R, Giladi N, Korczyn AD, Gurevich T, Sadeh M. Quantitative anal sphincter EMG in multisystem atrophy and 100 controls. J Neurol Neurosurg Psychiatry 2001;71(5):596–599.
9. Podnar S, Vodusek DB, Stålberg E. Comparison of quantitative techniques in anal sphincter electromyography. Muscle Nerve 2002;25(1):83–92.
10. Tison F, Arne P, Sourgen C, Chrysostome V, Yeklef F. The value of external anal sphincter electromyography for the diagnosis of multiple system atrophy. Mov Disord 2000;15(6):1148–1157.
11. Pramstaller PP, Wenning GK, Smith SJ, Beck RO, Quinn NP, Fowler CJ. Nerve conduction studies, skeletal muscle EMG, and sphincter EMG in multiple system atrophy. J Neurol Neurosurg Psychiatry 1995;58(5):618–621.
12. Stocchi F, Carbone A, Inghilleri M, et al. Urodynamic and neurophysiological evaluation in Parkinson's disease and multiple system atrophy. J Neurol Neurosurg Psychiatry 1997;62(5):507–511.
13. Lee EA, Kim BJ, Lee WY. Diagnosing multiple system atrophy with greater accuracy: combined analysis of the clonidine-growth hormone test and external anal sphincter electromyography. Mov Disord 2002;17(6):1242–1247.
14. Gilad R, Giladi N, Korczyn AD, Gurevich T, Sadeh M. Quantitative anal sphincter EMG in multisystem atrophy and 100 controls. J Neurol Neurosurg Psychiatry 2001;71(5):596–599.
15. Schwarz J, Kornhuber M, Bischoff C, Straube A. Electromyography of the external anal sphincter in patients with Parkinson's disease and multiple system atrophy: frequency of abnormal spontaneous activity and polyphasic motor unit potentials. Muscle Nerve 1997;20(9):1167–1172.
16. Linder J, Libelius R, Nordh E, Holmberg B, Stenlund H, Forsgren L. Anal sphincter electromyography in patients with newly diagnosed idiopathic parkinsonism. Acta Neurol Scand 2012;126(4):248–255.
