# Supplementary material for: Diagnostic and Prognostic Value of External Anal Sphincter EMG Patterns in Multiple System Atrophy
Source: Mov Disord. 2022 Feb 4;37(5):1069–74. doi: 10.1002/mds.28938 (PMC9305564; doi:10.1002/mds.28938)
Supplement: Supplementary file 2 — APPENDIX S2. Supporting Information [file MDS-37-1069-s003.docx]

**Demographic and clinical differences based on EAS EMG patterns in MSA**

In the MSA cohort, EAS EMG patterns were not associated with gender, age at symptom onset, age and disease duration at EMG, LEDD, UMSARS II score, or phenotype.

Instead, EAS EMG patterns were related to symptom type at onset in this group (*P* < 0.001) (Fig. 2A). Subjects with pattern I showed a prevalence of motor onset (87.5%), as opposed to urogenital disturbances (none, *P* < 0.001) or orthostatic symptoms at onset (12.5%, *P* = 0.001). Patients with pattern II showed a more even distribution of symptoms at onset (urogenital disturbances: 38.5%; orthostatic symptoms: 23%; motor impairment: 38.5%; *P* = 0.154). Instead, subjects with patterns III and IV more frequently showed urogenital disturbances at onset (70.0% and 87.5%, respectively), while fewer patients reported orthostatic symptoms at onset (13.3% of those with pattern III, *P =* 0.002; none with pattern IV, *P* < 0.001) or a motor onset (16.7% in the subgroup with pattern III, *P =* 0.003; 12.5% of patients with pattern IV, *P =* 0.001). The predominance of urogenital symptoms at onset in subjects with patterns III or IV, versus the group with pattern I, was significant (*P* < 0.001 and *P* = 0.002, respectively).

In MSA patients, an association was also found between EAS EMG patterns and the prevalence of urogenital symptoms and fecal incontinence at the time of EMG (Fig. 2B, C). Urogenital disturbances were uncommon in subjects with pattern I (12.5%, *P* < 0.001), frequent among patients with patterns II (73.1%, *P* = 0.011) or III (86.7%, *P* = 0.001), and were always present in subjects with pattern IV (*P* < 0.001) (Fig. 2B). Urogenital symptoms were preponderant in patients with patterns II, III or IV with respect to subjects with pattern I (*P* = 0.001, *P* < 0.001, and *P* = 0.002, respectively), and in patients with pattern IV as compared with subjects with pattern II (*P* = 0.008). Fecal incontinence was never reported by patients with pattern I (*P* < 0.001), whereas it was significantly present among subjects with patterns II (65.4%, *P* = 0.012) or III (76.7%, *P* = 0.009), and was always reported by patients with pattern IV (*P* < 0.001) (Fig. 2C). Fecal incontinence was predominant in patients with patterns II, III or IV as compared with subjects with pattern I (*P* = 0.001, *P* < 0.001, and *P* = 0.002, respectively), and in patients with pattern IV vs. pattern II (*P* = 0.008).
