# Supplementary figures and images for: Diagnostic and Prognostic Value of External Anal Sphincter EMG Patterns in Multiple System Atrophy
Source: Mov Disord. 2022 Feb 4;37(5):1069–74. doi: 10.1002/mds.28938 (PMC9305564; doi:10.1002/mds.28938)

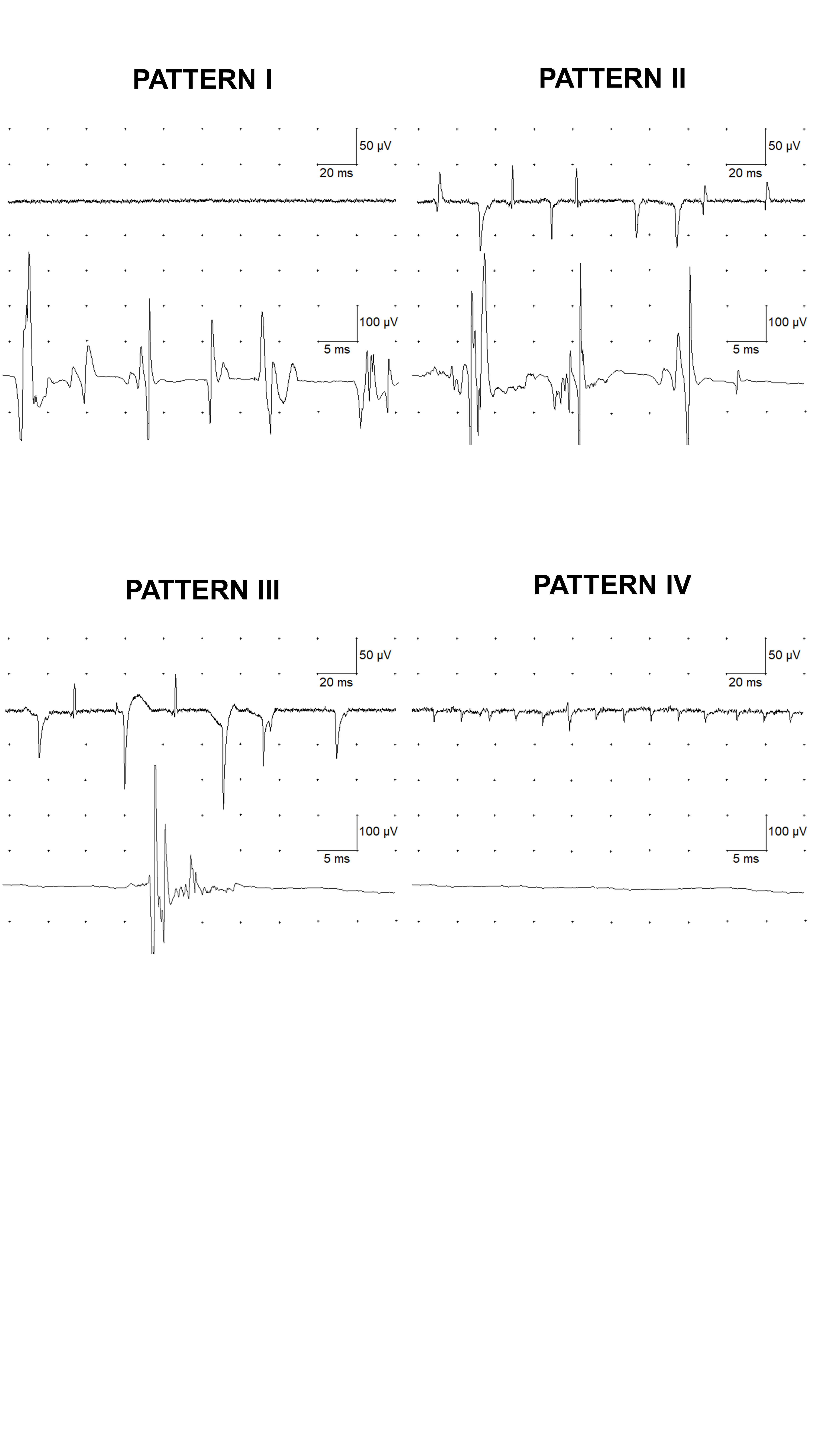

Supplement: Supplementary file 3 — FIG. S1 Illustrative traces of EAS EMG patterns. In each panel, the upper traces show the presence or absence of pathological spontaneous activity, whereas the lower traces show motor unit action potential (MUAP) parameters (ie, duration and spatial recruitment). Pattern I: no spontaneous activity and normal MUAP parameters. Pattern II: spontaneous activity (fibrillation potentials and positive sharp waves), increased duration of MUAPs, and normal recruitment of MUAPs (three MUAPs per insertion site). Pattern III: spontaneous activity (fibrillation potentials and positive sharp waves), increased duration of MUAPs, and reduced recruitment of MUAPs (one MUAP per insertion site). Pattern IV: spontaneous activity (complex repetitive discharges) and absent recruitment of MUAPs. EAS, external anal sphincter. [file MDS-37-1069-s002.tif]
