# Supplementary material for: Diagnostic and Prognostic Value of External Anal Sphincter EMG Patterns in Multiple System Atrophy
Source: Mov Disord. 2022 Feb 4;37(5):1069–74. doi: 10.1002/mds.28938 (PMC9305564; doi:10.1002/mds.28938)
Supplement: Supplementary file 4 — TABLE S1 Demographic and clinical features in MSA and PD patients [file MDS-37-1069-s004.docx]

**TABLE S1.** Demographic and clinical features in MSA and PD patients

|  |  |  | MSA  (n = 72) | PD  (n = 21) |
| --- | --- | --- | --- | --- |
| **Gender, male** | | | 35 (48.6) | 13 (61.9) |
| **Disease onset** | **Age, years** | | 63.9 ± 9.7 | 59.5 ± 11.4 |
|  | **Urogenital symptoms** | | 38 (52.8) | - |
|  | **Orthostatic symptoms** | | 11 (15.3) | - |
|  | **Motor symptoms** | | 23 (31.9) | - |
| **Hospital admission/EMG time** | **Age, years** | | 68.3 ± 9.6 | 64.9 ± 10.0 |
|  | **Disease duration, years** | | 4.4 ± 2.4 | 5.4 ± 4.1 |
|  | **LEDD, mg** | | 601.2 ± 447.1 | 681.4 ± 305.3 |
|  | **MDS-UPDRS III, score** | | - | 25.6 ± 9.5 |
|  | **UMSARS II, score** | | 19.9 ± 5.6 | - |
|  | **MSA-P phenotype** | | 60 (83.3) | - |
|  | **MSA-C phenotype** | | 12 (16.7) | - |
|  | **Urogenital symptoms** | | 54 (75.0) | - |
|  | **Fecal incontinence** | | 48 (66.7) | - |
|  | **Orthostatic symptoms** | | 49 (68.1) | - |

Data are reported as number of patients (%) or mean ± SD. The mean follow-up duration was 8.6 ± 3.4 years.
